# Supervision and assessment mechanisms and work performance among rural public servants: the mediating role of public service motivation and the moderating effect of red tape

**DOI:** 10.3389/fpsyg.2026.1815653

**Published:** 2026-09-24

**Authors:** Da-Peng Xu, Xin-Hui Qiu, Qing-Zhi Jiang, Meng-Lu Zhu

**Affiliations:** 1 School of History and Culture, Qufu Normal University, Qufu, Shandong, China; 2 Faculty of Psychology, Southwest University, Chongqing, China; 3 School of Public Administration, Huazhong Agricultural University, Wuhan, Hubei, China

**Keywords:** moderated mediation, public service motivation, red tape, supervision and assessment mechanisms, work performance

## Abstract

**Background:**

Village-assigned officials play a pivotal role in advancing rural revitalization and implementing policy at the grassroots level. Drawing on Conservation of Resources (COR) theory, this study investigates how supervision and assessment mechanisms (SAM) affect work performance by examining the mediating role of public service motivation (PSM) and the moderating effect of red tape. By integrating motivational and institutional perspectives, this study provides new insights into the mechanisms underlying performance management in public administrations.

**Methods:**

Data were collected from 354 village-assigned officials in two counties and two county-level cities under the jurisdiction of City C in Shandong Province, China, through a formal online survey conducted between May and June 2026. Mediation, moderation, and moderated mediation analyses were conducted using Mplus.

**Results:**

Supervision and assessment mechanisms had a significant influence on PSM, which in turn positively affected the level of work performance. Red tape moderated the effect of supervision and assessment mechanisms on PSM. Specifically, moderated mediation analysis demonstrated that red tape moderated the positive indirect effect of supervision and assessment mechanisms on work performance through PSM so that the indirect effect decreased with increasing red tape.

**Conclusion:**

Supervision and assessment mechanisms significantly improve rural public servants’ work performance, with public service motivation serving as a key mediator. However, red tape undermines these motivational benefits. Public organizations should strive to attenuate the degree of red tape that public servants feel and experience to elicit public service motivation, thereby enhancing work performance and strengthening grassroots governance effectiveness.

## 1 Introduction

The implementation of China’s Rural Revitalization Strategy has placed increasing emphasis on strengthening grassroots governance and improving the performance of village-assigned officials. As an important institutional arrangement for promoting rural development, village-assigned officials undertake multiple responsibilities, including policy implementation, industrial development, public service delivery, and rural governance. Their work performance directly influences the effectiveness of rural revitalization policies and the modernization of grassroots governance. Consequently, identifying the factors that enhance the work performance of village-assigned officials has become an important issue in both public administration research and management practice.

Previous studies have consistently identified supervision and assessment mechanisms (SAM) as important managerial instruments for improving employee performance. Drawing on goal-setting theory, feedback intervention theory, and social exchange theory, scholars have argued that effective supervision and assessment mechanisms clarify organizational expectations, strengthen accountability, provide performance feedback, and ultimately improve employee performance (Locke and Latham, 1990; Carver and Scheier, 1982; Homans, 1958; DeNisi and Pritchard, 2006). In China’s public sector, supervision and assessment systems have become increasingly institutionalized and are widely regarded as essential tools for promoting administrative efficiency and policy implementation. However, empirical evidence also suggests that supervision and assessment do not always produce consistent performance outcomes. While some studies report significant motivational and performance improvements, others indicate that excessive assessment pressure or rigid performance indicators may reduce employees’ enthusiasm and weaken policy implementation (Mao, 2018). These inconsistent findings imply that the effectiveness of supervision and assessment mechanisms may depend on both employees’ psychological responses and the organizational context in which these mechanisms are implemented.

Public service motivation (PSM) has frequently been proposed as an important psychological mechanism linking management practices to employee performance. Defined as an individual’s predisposition to serve the public interest and pursue collective welfare (Perry and Wise, 1990), PSM has consistently been associated with higher work engagement, organizational commitment, and work performance. Accordingly, previous studies have increasingly examined the mediating role of PSM in explaining how supervision and assessment mechanisms improve work performance (Perry, 1996; Jung, 2014; Wen and Tao, 2022). Although this line of research has substantially advanced understanding of the motivational mechanism underlying performance improvement, it primarily explains how supervision and assessment mechanisms influence employee performance, while paying relatively limited attention to when and under what organizational conditions this motivational process becomes stronger or weaker.

One organizational factor that may explain these differences is red tape. Red tape refers to burdensome rules, procedures, and administrative formalities that impose compliance costs without proportionate contributions to organizational objectives (Bozeman and Feeney, 2014; Pandey and Scott, 2002). Previous studies and meta-analyses have consistently demonstrated that red tape negatively affects employee attitudes, work motivation, organizational commitment, and organizational performance (Blom et al., 2021; George et al., 2021). However, existing studies have primarily examined the direct effects of red tape on employee or organizational outcomes. Comparatively less attention has been devoted to examining whether red tape functions as an organizational contextual factor that constrains the effectiveness of management practices. In particular, little empirical research has investigated whether red tape weakens the motivational process through which supervision and assessment mechanisms enhance public service motivation and subsequently improve work performance. This issue is especially important in the context of village-assigned officials, whose daily work is characterized by intensive performance assessment while simultaneously involving substantial administrative burdens, repetitive reporting requirements, and procedural compliance.

To address these limitations, this study develops a moderated mediation model grounded in Conservation of Resources (COR) theory (Hobfoll, 1989). COR theory posits that individuals strive to obtain, preserve, and protect valuable resources, and that work motivation and performance are influenced by the availability or depletion of these resources. In the present study, supervision and assessment mechanisms are conceptualized as organizational resources that enhance public service motivation, whereas red tape represents a contextual constraint that consumes employees’ psychological resources and weakens the motivational benefits generated by supervision and assessment mechanisms. Although the Job Demands–Resources (JD-R) model provides a complementary perspective for understanding how job demands influence employee outcomes, it is conceptually rooted in COR theory and therefore serves only as a supporting framework rather than the primary theoretical foundation of this study. Specifically, public service motivation is proposed as the psychological mechanism through which supervision and assessment mechanisms influence work performance, whereas red tape is expected to moderate the relationship between supervision and assessment mechanisms and public service motivation. Unlike previous studies that primarily focused on the independent effects of supervision and assessment mechanisms, public service motivation, or red tape, the present study further examines whether red tape serves as a critical boundary condition that weakens the transformation of supervision and assessment mechanisms into public service motivation, thereby influencing subsequent work performance. By integrating these relationships within the framework of Conservation of Resources (COR) theory, this study contributes to the literature in three ways. First, it extends research on supervision and assessment mechanisms by incorporating organizational context into the supervision–motivation–performance process. Second, it enriches the literature on public service motivation by identifying the organizational conditions under which its motivational effect is strengthened or constrained. Third, it provides new evidence from village-assigned officials in China, offering practical implications for optimizing cadre assessment systems and improving grassroots governance capacity.

## 2 Theoretical background and hypotheses

### 2.1 The mediation effect of supervision and assessment mechanisms

Public service motivation (PSM) refers to an individual’s predisposition to respond to motives grounded primarily or uniquely in public institutions and organizations (Perry and Wise, 1990). Employees with high levels of PSM are intrinsically motivated to serve the public interest and contribute to society rather than merely pursuing material rewards. Consequently, PSM has been widely recognized as a distinctive motivational resource in public organizations because it encourages employees to devote greater effort to their work, persist when facing difficulties, and actively pursue organizational and societal goals (Perry et al., 2010; Ritz et al., 2016). Recent systematic reviews have further confirmed that PSM remains one of the most influential motivational constructs in public administration and consistently predicts a wide range of positive employee attitudes and behaviors across different institutional contexts (Chung et al., 2024).

Supervision and assessment mechanisms constitute an essential component of personnel management in public organizations. Rather than functioning solely as administrative control tools, effective supervision and assessment systems communicate organizational expectations, provide performance feedback, and reinforce desirable work behaviors. According to Social Cognitive Theory (Bandura, 1986), employees continuously interpret information from their organizational environment and transform external experiences into internal cognitive beliefs that subsequently guide their motivation and behavior. Fair and transparent supervision enables employees to recognize organizational justice and accountability, while objective performance assessment provides evidence that their efforts are valued and recognized. These organizational cues strengthen employees’ confidence in accomplishing public responsibilities and reinforce the perception that their work contributes meaningfully to society. As employees gradually internalize these values, they become increasingly motivated to serve the public, thereby enhancing their public service motivation. Previous studies have similarly suggested that supportive organizational environments, procedural fairness, and developmental human resource management practices are important antecedents of PSM (Vandenabeele, 2007; Wright and Grant, 2010).

Public service motivation has consistently been identified as an important predictor of work performance in public organizations. Employees with stronger PSM are more likely to devote discretionary effort to task accomplishment, maintain persistence under demanding working conditions, and voluntarily engage in behaviors that improve service quality and organizational effectiveness. Unlike external incentives that primarily influence behavior through material rewards, PSM encourages employees to pursue organizational objectives because they perceive public service as personally meaningful and socially valuable. Such intrinsic motivation promotes greater initiative, responsibility, and sustained work engagement, ultimately translating into superior work performance. Extensive empirical evidence, including recent systematic reviews and meta-analytic studies, has consistently demonstrated that employees with higher levels of PSM achieve better individual performance and make greater contributions to organizational effectiveness (Bright, 2007; Kim, 2004; Ritz et al., 2016; Ritz et al., 2021).

Although supervision and assessment mechanisms can directly improve work performance by clarifying responsibilities and strengthening accountability, their influence is unlikely to rely solely on external control. From the perspective of Social Cognitive Theory, organizational environments affect employee behavior primarily by shaping cognitive and motivational processes rather than determining behavior directly (Bandura, 1986). Employees who perceive supervision and assessment mechanisms as fair, transparent, and developmental are more likely to internalize organizational values, strengthen their commitment to public service, and develop higher levels of public service motivation. Enhanced PSM subsequently encourages employees to invest greater effort, maintain stronger persistence, and continuously improve the quality of public service, ultimately resulting in higher work performance. Therefore, public service motivation is expected to function as an important psychological mechanism linking supervision and assessment mechanisms to work performance.

Therefore, the following mediation hypothesis is proposed.

*Hypothesis 1*: The effect of supervision and assessment mechanisms on work performance is mediated by public service motivation.

### 2.2 The moderating role of red tape

Red tape is a “role-specific subjective experience of compliance burden imposed by an organization (Pandey, 2021, p. 264). A psychological process aspect of red tape make employees in the organization perceive a compliance burden. A recent meta-analysis (Blom et al., 2021) revealed a negative relationship between red tape and public servants’ attitudes and behavior. In this meta-analysis, red tape has a negative impact on employee-level outcomes (perceived autonomy, commitment, work motivation, and work satisfaction, etc.) and organization-level outcomes (organizational support, goal clarity, communication, innovation, etc.). Another meta-analysis (George et al., 2021) also showed that red tape had a significant, negative effects on both organizational performance and employee outcomes.

Conservation of Resources (COR) theory (Hobfoll, 1989) provides the primary theoretical framework for explaining how red tape influences public service motivation. The Job Demands–Resources (JD-R) model is conceptually consistent with COR theory and provides a complementary work-context perspective by distinguishing between job demands and job resources. The JD-R model contends that every occupation has two characteristics: job demands and job resources. Job demands (e.g., workload, cognitive demands, etc.) relate to the aspects that require continuous physical or mental efforts. Meanwhile, job resources (e.g., decision latitude, social support, etc.) pertain to the aspects that facilitate work goals and personal growth. The JD-R model assumes two processes: the energy depletion process and the motivational process. According to the energy depletion process, job demands exhaust employees’ mental and physical resources and lead to energy depletion. Through the motivational process, resources, due to their motivational role, stimulate work engagement and positive job outcomes.

COR theory posits that employees tend to strive to acquire, protect and retain valuable resources and do their best to avoid resource loss. Within the JD-R framework, red tape can be viewed as a hindrance job demand because it imposes unnecessary compliance burdens on employees. Consistent with COR theory, such hindrance demands consume valuable psychological resources and increase the likelihood of resource loss (Fuenzalida et al., 2026).

According to COR theory, employees experiencing persistent resource loss are more likely to adopt defensive strategies to conserve their remaining resources (Hobfoll et al., 2018). Because red tape consumes emotional and cognitive resources, employees have fewer resources available to sustain prosocial motivation, thereby weakening public service motivation. Employees who experience negative emotions tend to have fewer emotional resources. In addition, red tape depletes individuals’ emotional resources (Qiang et al., 2025). High levels of red tape presents a considerable compliance burden that public servants have to comply with, and it requires employees to expend more resources (Huang et al., 2022). COR theory contends that employees who suffer resource-depleting situations tend to engage in defensive behaviors (Hobfoll et al., 2018), leading them to the reduction of employees’ PSM behavior. Employees’ efforts to reduce the loss of emotional resources severely affects the level of PSM. That is, red tape increases negative emotions, thereby inhibiting PSM. In empirical research (Moynihan and Pandey, 2007), red tape significantly buffered the positive relationship between SAM and PSM. In this regard, Moynihan and Pandey (2007) explained that red tape discouraged employees from believing they are serving the public good. This means that red tape will reduce the positive effect of SAM on PSM. Based on the above background, the following moderation hypothesis is proposed.

*Hypothesis 2*: Red tape moderates the positive relationship between SAM and PSM such that this relationship is weaker when red tape is higher.

### 2.3 The moderated mediation analysis

The relationship between SAM and PSM can be analyzed in relation to two-way interaction and the hypothesized model includes work performance as the final dependent variable. On a philosophical level, “all effects are mediated by something” (Hayes, 2022, p. 127). Likewise, “it is safe to say that all effects are moderated by something” (Hayes, 2022, p. 410). In line with this reasoning, the present study established an integrated moderated mediation model which combined the above simple mediation model and simple moderation model. Moderated mediation model means that the positive indirect effect of SAM on work performance through PSM is dependent upon the value of red tape. In particular, the indirect effect of SAM is minimized when the value of red tape is high. That is, the indirect effect is strengthened at lower levels of red tape. Taken together, we formulated the following hypothesis.

*Hypothesis 3*: Red tape moderates the indirect effect of SAM on work performance via PSM, so that when red tape is high, the indirect effect becomes weaker.

The conceptual model corresponding to the above hypothesized process is presented in Figure 1. The model means that the indirect effect of SAM on work performance through PSM is moderated by red tape.

**Figure 1:**
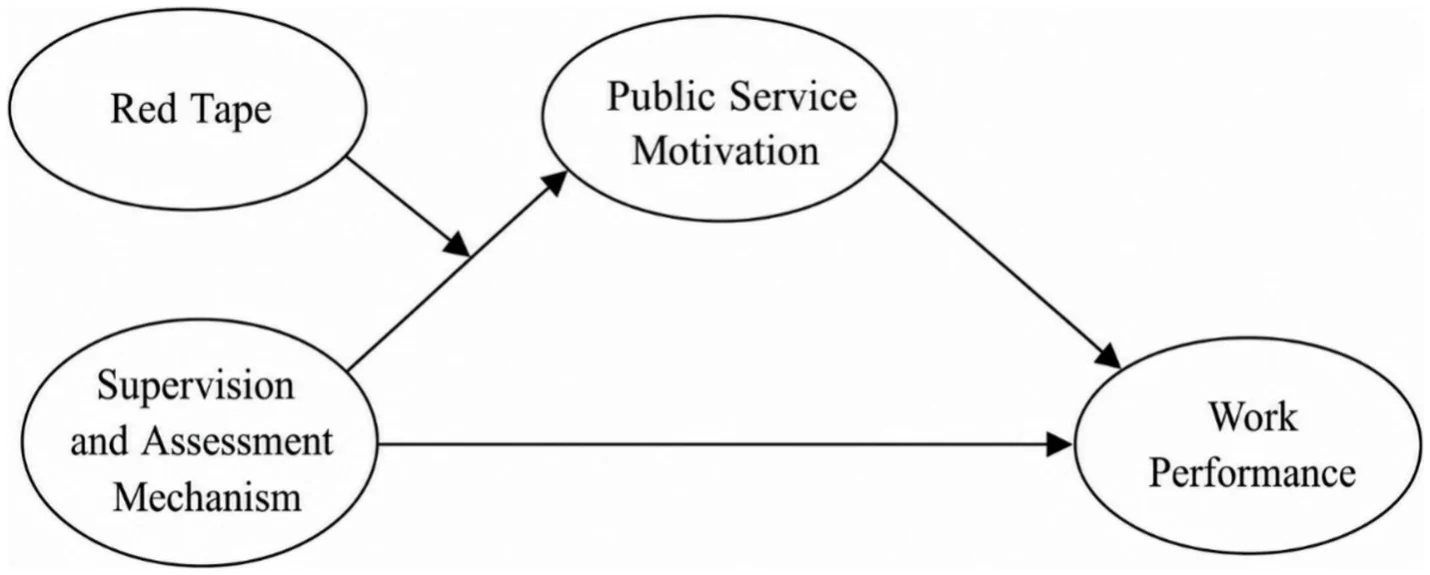
The moderated mediation model.

## 3 Methodology

### 3.1 Participants and procedure

The participants were village‐assigned officials from the two counties and two county‐level cities under the jurisdiction of City C in Shandong Province, China. City C administers two districts, two counties, and two county‐level cities, covering 62 towns, six townships, and 3,577 administrative villages. Village‐assigned officials in this region are primarily deployed in county‐level towns, townships, and administrative villages.

An initial round of data collection was conducted between December 2023 and January 2024; however, the initial dataset suffered from notable data flaws and unsatisfactory reliability, making it unsuitable for formal hypothesis testing. Therefore, a second‐round formal survey was carried out between May 2026 and June 2026. Data for the present study were collected in this second‐round formal survey. With the assistance of grassroots Party organizations, the questionnaire was distributed to village‐assigned officials working in the selected counties and county‐level cities. A total of 402 questionnaires were returned. After 48 invalid questionnaires were excluded, 354 valid responses were retained for the final analysis, yielding an effective response rate of 88.06%.

The demographic characteristics of the participants are presented in Table 1.

**Table 1:** Frequency analysis results.

Variable	Category	Frequency	Percentage (%)
Gender (*n* = 354)	Male	240	67.8
	Female	114	32.2
Age (*n* = 354)	18–30	113	31.9
	31–40	108	30.5
	41–50	82	23.2
	51–60	51	14.4
Education (*n* = 354)	Associate	25	7.1
	Bachelor	223	63.0
	Master or above	106	29.9
Job type (*n* = 354)	Public welfare	96	27.1
	Institutional	19	5.4
	Administrative	190	53.7
	Other	49	13.8
Position (*n* = 354)	First-level clerk	180	50.8
	Second-/first-level principal clerk	109	30.8
	Fourth-/third-level principal clerk	31	8.8
	Other	34	9.6
Total		354	100

### 3.2 Measures

The questionnaire included demographic information and measures of supervision and assessment mechanisms, public service motivation, work performance, and red tape. The demographic variables included gender, age, educational level, job type, and position. All substantive variables were assessed using a seven-point Likert scale ranging from 1 (“strongly disagree”) to 7 (“strongly agree”). Higher scores indicated higher levels of the corresponding constructs. The wording of the original measurement items was refined through pilot testing to improve its clarity and suitability for the work context of village-assigned officials in China.

Although many organizational studies employ measurement scales developed in Western contexts, previous methodological research has emphasized that cross-cultural measurement requires conceptual and contextual equivalence rather than simple linguistic translation (Gjersing et al., 2010). Moreover, Farh et al. (2006) suggested that context-specific measures may provide higher construct validity when the focal constructs are strongly shaped by indigenous institutional environments. Given that supervision and assessment mechanisms are closely associated with China’s public personnel management system, this study adopted measurement scales developed and validated in the Chinese public-sector context to better capture the institutional characteristics of the construct.

#### 3.2.1 Supervision and assessment mechanisms

Supervision and assessment mechanisms were measured using a six-item scale adapted from measures developed and applied in Chinese organizational and public-sector settings (Han et al., 2007; Jia, 2010; Li, 2023; Su, 2023; Zhou, 2018). The six items assessed goal orientation, assessment indicators, weight distribution, goal communication, result feedback, and result application.

Chinese domestic measures were adopted because supervision and assessment mechanisms are embedded in China’s distinctive administrative and institutional context. In particular, the supervision of village-assigned officials is characterized by hierarchical accountability, target-based management, performance assessment, and the organizational leadership of local Party and government authorities. Measures developed in other institutional contexts may not fully capture these context-specific characteristics. Using locally developed and validated measures therefore helped enhance the contextual and cultural validity of the construct.

#### 3.2.2 Public service motivation

Public service motivation was measured using a four-item scale adapted from Perry’s public service motivation framework and its subsequent applications in the Chinese public-sector context (Bao and Li, 2016; Guo and Guo, 2019; Han, 2021; Li and Wang, 2016). The four items represented attraction to policy making, commitment to the public interest, self-sacrifice, and compassion. The wording of the items was adjusted to reflect the public service responsibilities of village-assigned officials. Higher scores indicated stronger public service motivation.

#### 3.2.3 Work performance

Work performance was measured using a three-item scale adapted from measures previously applied in Chinese public-sector research (Liao, 2021; Yu, 2023; Zhang, 2022; Wang, 2023). The three items assessed dedication, interpersonal facilitation, and task performance. Minor wording modifications were made to ensure that the items were appropriate for village-assigned officials. For example, general references to “colleagues” were replaced with “village-assigned officials” where necessary. Higher scores indicated better work performance.

#### 3.2.4 Red tape

Red tape was measured using a three-item scale adapted from Zhao (2022) and the conceptualization of organizational rules and red tape developed by Bozeman and Feeney (2014). The items assessed the extent to which formal procedures, information-sharing rules, and organizational regulations restricted village-assigned officials’ access to necessary information and their ability to complete work tasks effectively. The wording was adjusted to fit the village-governance context in China. Higher scores indicated higher perceived levels of organizational red tape.

## 4 Results

### 4.1 Correlation analysis

Means and inter-correlations among study variables are presented in Table 2. As shown in Table 2, work performance is positively correlated with public service motivation (*r* = 0.611, *p* < 0.001) and supervision and assessment mechanisms (*r* = 0.621, *p* < 0.001). Public service motivation is positively correlated with supervision and assessment mechanisms (*r* = 0.685, *p* < 0.001). In terms of red tape, the construct is negatively correlated with work performance (*r* = −0.562, *p* < 0.001), public service motivation (*r* = −0.861, *p* < 0.001) and supervision and assessment mechanisms (*r* = −0.615, *p* < 0.001). The mean score of the study variables ranged from 3.67 to 4.43.

**Table 2:** Descriptive statistics and correlations.

Variables	Mean	SD	1	2	3	4
1 Work performance	4.214	1.140	–			
2 Public service motivation	3.665	1.053	0.611	–		
3 Supervision and assessment mechanisms	4.349	1.029	0.621	0.685	–	
4 Red tape	4.426	1.449	−0.562	−0.861	−0.615	–

Correlations ≥|0.562| are significant at the 0.001 level or better (two-tailed test).

### 4.2 Confirmatory factor analysis

We conducted a confirmatory factor analysis (CFA) using Mplus SEM software to validate the measurement model. Except for red tape, the value of each dimension for three factors (SAM, PSM, work performance) is calculated using average values of respective three measurement items. Each latent construct was represented by multiple dimensions, and the indicators entered into the CFA were the average scores of the items within each dimension rather than individual questionnaire items. Accordingly, SAM was represented by six indicators derived from 18 items, PSM by four indicators derived from 12 items, work performance by three indicators derived from nine items. Therefore, the measurement model consisted of four latent constructs and 16 indicators.

To assess the model fit, we used the root means square error of approximation (RMSEA), the comparative fit index (CFI), the Tucker-Lewis index (TLI) and the standardized root mean square residual (SRMR). The results revealed that the hypothesized four-factor model fit the data very well (*χ*^2^ (*df* = 98) = 90.241, *p* = 0.699, CFI = 1.000, TLI = 1.006, RMSEA = 0.000, SRMR = 0.030). Construct validity is the extent to which a set of indicators actually reflects the latent construct these indicators are designed to measure (Hair et al., 2010). Construct validity consists of two components, namely convergent validity and discriminant validity. Composite reliability (CR) and average variance extracted (AVE) were used to verify convergent validity. The CR of each variable was above the recommended threshold of 0.70 and the AVE of each variable met the typical benchmark level of 0.50 (Hair et al., 2010), suggesting that the measurement model obtained sufficient convergent validity. Additionally, the square root of each AVE value is greater than its correlations with other constructs, implying that the discriminant validity was satisfied (Fornell and Larcker, 1981). Another criterion for discriminant validity is that intercorrelations among a set of factors should be less than 0.90 (Kline, 2016). All the correlations were less than 0.90, supporting the evidence of discriminant validity. The results of confirmatory factor analysis is presented in Table 3.

**Table 3:** Factor loadings, Cronbach’s alpha, composite reliability, average variance extracted.

Construct	Items	Factor loading	Cronbach’s alpha	CR	AVE
SAM	SAM1	0.756	0.875	0.899	0.597
	SAM2	0.775			
	SAM3	0.715			
	SAM4	0.768			
	SAM5	0.717			
	SAM6	0.723			
PSM	PSM1	0.867	0.923	0.932	0.774
	PSM2	0.895			
	PSM3	0.854			
	PSM4	0.835			
WP	WP1	0.765	0.845	0.870	0.691
	WP2	0.826			
	WP3	0.834			
Red tape	RT1	0.887	0.912	0.920	0.794
	RT2	0.865			
	RT3	0.896			

CR, composite reliability; WP, work performance.

### 4.3 Common method bias

Given that our data were collected from a single source, an assessment for the common method bias (CMB) was made using Harman’s one-factor test (Podsakoff et al., 2003). The underlying CMB among the study variables may cause a serious problem due to its impact on the correlations of variables. Traditionally, Harman’s one-factor test indicates problematic CMB if unrotated principal-component factor analysis produces eigenvalues suggesting the first factor accounts for more than 50% of the variance among the measures. Harman’s one-factor test was conducted to determine whether a substantial amount of CMB is present. Because the variance of the first factor accounted for only 31% of the total variance, CMB is not a great concern in the present study.

### 4.4 Indirect effect of SAM

To test whether the effect of SAM on work performance is mediated by PSM, we performed mediation analysis using Mplus. The proposed mediation model fits the data very well (*χ*^2^ (*df* = 62) = 49.477, *p* = 0.875, RMSEA = 0.000, CFI = 1.000, TLI = 1.014, SRMR = 0.025). Table 4 shows the results of mediation analysis. As shown in Table 4, SAM is positively related to PSM (*a* = 1.036, *p* < 0.001) and PSM is positively related to work performance (*b* = 1.140. *p* < 0.001).

**Table 4:** Model coefficients.

Antecedent	Consequent					
	PSM			Work performance		
	Estimate	SE	*p*	Estimate	SE	*p*
SAM	1.036	0.128	0.000	−0.663	0.284	0.020
PSM	–	–	–	1.140	0.260	0.000

The Sobel test results indicate that the indirect effect is positive and significant (*ab* = 1.181, *p* < 0.001). Such results show that PSM functions as a mediator of the effect of SAM on work performance. To further investigate the mediation effect, we constructed 95% bootstrap confidence interval for the indirect effect, based on 10,000 bootstrap samples. Unlike the Sobel test, bootstrap confidence interval method does not assume that the sampling distribution of the indirect effect is normal.

Specifically, the bootstrap confidence interval method tends to have higher power than the Sobel test (Hayes, 2022). The absence of zero in the 95% bootstrap confidence interval for the estimate indicates that the indirect effect is statistically different from zero. As 95% confidence interval is entirely above zero (0.772–1.915), there is clear evidence that the indirect effect is positive. These findings reveal that Chinese public servants’ SAM positively affects their PSM, which in turn positively affects their level of work performance. Thus, hypothesis 1 was supported.

### 4.5 Moderation analysis

Using Mplus, we tested whether red tape moderates the effect of SAM on PSM. Prior to moderation analysis, all variables were mean-centered.

If the interaction between SAM and red tape (SAM × red tape: *b*_3_) is statistically different from zero, we can conclude that the effect of SAM on PSM is moderated by red tape (Hayes, 2022). As presented in Table 5, the interaction coefficient for SAM and red tape was negatively significant (*b*_3_ = −0.149, *p* < 0.01), implying that SAM has a different effect on PSM depending on the value of red tape. When moderation effect is significant, Hayes (2022) suggested probing an interaction to ascertain where in the distribution of the moderator (red tape: *W*) SAM has an effect on PSM that is different from zero and where it does not.

**Table 5:** Results of moderation analysis.

Variables	Estimate	SE	*t*	*p*
SAM	1.033	0.128	8.057	0.000
Red tape	−0.055	0.034	−1.622	0.105
SAM × red tape	−0.149	0.050	−2.979	0.003

A typical strategy when probing an interaction is to estimate the conditional effect of SAM on PSM when red tape is equal to the mean ( $\bar{W}$ ), a standard deviation below the mean ( $\bar{W}-SD_{W}$ ), and a standard deviation above the mean ( $\bar{W}+SD_{W}$ ). We reported the results of probing in Table 6. As shown in Table 6, the positive effect of SAM on PSM decreases as the value of red tape increases. The positive relationship between SAM and PSM is strengthened when red tape is low (simple slope = 1.182, *p* < 0.001) compared to high (simple slope = 0.885, *p* < 0.001). That is, red tape moderated the positive relationship between SAM and PSM, so that this relationship became weaker as red tape increased. Therefore, hypothesis 2 was supported.

**Table 6:** Results of probing (simple slope analysis).

Value of red tape	Estimate	SE	*t*	*p*
$\bar{W}-SD_{W}$	1.182	0.155	7.604	0.000
$\bar{W}$	1.033	0.128	8.057	0.000
$\bar{W}+SD_{W}$	0.885	0.117	7.552	0.000

### 4.6 Moderated mediation analysis

Thus far, moderation analysis and mediation analysis have been treated as distinct concepts with different analytical processes. Now we tested moderated mediation model which integrates mediation model and moderation model. A moderated mediation model posits that moderator (red tape) would weaken or strength the indirect effect of independent variable (SAM) on dependent variable (work performance) via mediator (PSM). Klein and Moosbrugger (2000) developed the latent moderated structural equations method (LMS) to estimate latent variable interactions in structural equation modeling (SEM) and the LMS can be easily implemented in Mplus. SEM is widely utilized to estimate structural parameters since SEM incorporates measurement errors into the analysis. Unlike product indicator approaches, the LMS (distribution analytic approach) accounts for nonlinearity in estimating parameters (Sardeshmukh and Vandenberg, 2017). “Of all the methods, the LMS method may be the most precise because it explicitly estimates the degree of non-normality” (Kline, 2016, p. 443).

Cheung and Lau (2017) suggested a three-step procedure for testing moderated mediation in the LMS method with Mplus. We followed Cheung and Lau’s (2017) procedure to test moderated mediation which includes latent variables interaction. The first step is to run a baseline model where the moderator is included, but latent interaction term is not included.

The baseline model provides adequate fit (*χ*^2^ (*df* = 99) = 92.941, *p* = 0.653, RMSEA = 0.000, CFI = 1.000, TLI = 1.004, SRMR = 0.032) and the factor loadings are statistically significant. Therefore, we can conclude that the indicators measure the constructs well and proceed to the next step.

In the second step, moderated mediation model (the model with the latent interaction term) is estimated. A test of moderated mediation is conducted by testing whether the index of moderated mediation is different from zero (Hayes, 2022). A test as to whether the index of moderated mediation is statistically significant serves as a formal test of moderated mediation (Hayes, 2015). The results of moderated mediation analysis by Mplus are shown in Table 7. As presented in Table 7, the index of moderated mediation is statistically significant (*a*_3_*b* = −0.133, *p* < 0.05), leading to the conclusion that the indirect effect of SAM on work performance through PSM is dependent upon the value of red tape. In the third step, we estimated the moderated mediation effect of SAM on work performance at various levels of red tape to probe the moderated mediation effect. We can substitute five levels of red tape in the equation that defines moderated mediation effect, as follows: (1) mean plus two standard deviations, (2) mean plus one standard deviation, (3) mean, (4) mean minus one standard deviation, (5) mean minus two standard deviations. Probing moderation of mediation is implemented by using the conditional indirect effect of SAM on work performance via PSM. The conditional indirect effects at various levels of red tape are presented in Table 8.

**Table 7:** Results of moderated mediation.

Variables	Estimate	SE	*t*	*p*
Outcome variable: PSM				
SAM	1.017	0.120	8.498	0.000
Red tape	−0.028	0.035	−0.787	0.431
SAM × red tape (*a*_3_)	−0.123	0.055	−2.234	0.025
Outcome variable: work performance				
SAM	−0.581	0.214	−2.717	0.007
PSM (*b*)	1.077	0.205	5.249	0.000

Index	Estimate	SE	*t*	*p*
Index of moderated mediation				
*a*_3_ *b*	−0.133	0.054	−2.472	0.013

**Table 8:** Probing moderated mediation (conditional indirect effect).

Value of red tape	Estimate	SE	*t*	*p*
$\bar{W}-2SD_{W}$	1.346	0.268	5.015	0.000
$\bar{W}-SD_{W}$	1.221	0.247	4.947	0.000
$\bar{W}$	1.095	0.234	4.681	0.000
$\bar{W}+SD_{W}$	0.970	0.231	4.190	0.000
$\bar{W}+2SD_{W}$	0.845	0.240	3.525	0.000

Table 8 shows that red tape changes the strength of the conditional indirect effect of SAM on work performance. Although the positive indirect effect of SAM on work performance through PSM is significant at all levels of red tape, the indirect effect decreased with increasing red tape. Hence, hypothesis 3 was supported.

## 5 Discussion

Based on data collected from 354 village-assigned officials in China, this study examined whether public service motivation (PSM) mediates the relationship between supervision and assessment mechanisms (SAM) and work performance, and whether red tape moderates this indirect relationship. The results supported the proposed moderated mediation model. Specifically, PSM mediated the positive relationship between SAM and work performance, whereas red tape negatively moderated the relationship between SAM and PSM. Furthermore, the indirect effect of SAM on work performance through PSM became progressively weaker as perceived red tape increased. These findings extend recent research on red tape by suggesting that its influence extends beyond directly affecting employee attitudes and organizational outcomes to shaping the effectiveness of organizational management practices. Recent research has emphasized that red tape should be understood as a psychological process arising from compliance burden rather than merely as a structural feature of bureaucracy (Fuenzalida et al., 2026). Building on this perspective, the present study further demonstrates that red tape weakens the process through which supervision and assessment mechanisms are translated into public service motivation, thereby reducing their indirect contribution to work performance.

The mediating role of PSM provides insight into the psychological process through which supervision and assessment arrangements are associated with public servants’ work performance. SAM not only establishes performance expectations and evaluation standards but also communicates which organizational goals and public values are considered important. When village-assigned officials perceive these mechanisms as meaningful and supportive, they are more likely to internalize organizational goals and develop stronger motivation to serve the public. This motivation, in turn, is associated with greater effort, persistence, and commitment in the performance of their duties. Thus, the effectiveness of SAM cannot be understood solely in terms of external monitoring and control; its motivational function is also important.

The negative moderating effect of red tape demonstrates that the effectiveness of supervision and assessment mechanisms depends not only on the design of the management system itself but also on the broader administrative environment in which it operates. Previous studies have primarily examined the direct effects of red tape on employee attitudes, work motivation, and organizational performance. The present findings provide a complementary perspective by showing that red tape also constrains the motivational effectiveness of supervision and assessment mechanisms. When village-assigned officials experience excessive reporting requirements, repetitive documentation, and procedural compliance, considerable cognitive and emotional resources are diverted toward fulfilling administrative obligations rather than substantive public service. Under these circumstances, supervision and assessment mechanisms are less likely to be interpreted as developmental guidance or organizational recognition and more likely to be perceived as additional administrative burdens. Consequently, even well-designed supervision and assessment mechanisms become less capable of strengthening public service motivation. This interpretation is consistent with recent research suggesting that compliance burdens associated with red tape primarily operate through psychological processes that gradually erode employees’ motivational resources (Fuenzalida et al., 2026).

The moderated mediation result further shows that the indirect effect of SAM on work performance through PSM is not equally strong for all village-assigned officials. The indirect effect was strongest when red tape was low and weakened as red tape increased. This finding suggests that reducing unnecessary administrative burdens is not merely an efficiency issue; it is also a necessary condition for transforming institutional arrangements into employee motivation and improved performance. Public organizations must therefore consider both the design of assessment mechanisms and the administrative conditions under which these mechanisms operate.

## 6 Theoretical implications

These findings have important theoretical implications. First, this study extends previous research on supervision and assessment mechanisms by demonstrating that their effectiveness depends not only on their design but also on the organizational context in which they are implemented. Second, whereas previous studies have primarily examined the direct effects of red tape on employee attitudes, wellbeing, motivation, and organizational outcomes, the present study identifies red tape as a critical organizational boundary condition influencing the motivational pathway from supervision and assessment mechanisms to work performance. Rather than directly determining employee performance, red tape weakens the transformation of supervision and assessment mechanisms into public service motivation, thereby reducing their indirect contribution to work performance. Finally, these findings complement recent studies emphasizing the psychological consequences of compliance burden (Fuenzalida et al., 2026) by further demonstrating that red tape also shapes the effectiveness of organizational human resource management practices.

## 7 Practical implications

The findings also have several practical implications for public organizations. First, managers should design supervision and assessment mechanisms that provide clear goals, reasonable evaluation criteria, effective communication, timely feedback, and meaningful use of assessment results. Assessment should not be limited to monitoring compliance. It should also help village-assigned officials understand how their work contributes to public interests and rural development.

Second, public organizations should integrate the cultivation of PSM into performance management. Managers can strengthen PSM by emphasizing the public value of village work, recognizing employees’ contributions, providing opportunities to participate in goal setting, and connecting individual responsibilities with broader community outcomes. Training and incentive programs should therefore address both employees’ professional capabilities and their commitment to public service.

Third, reducing red tape is essential for ensuring that SAM achieves its intended motivational effects. Public organizations should regularly review existing reporting requirements, approval procedures, documentation tasks, and information-sharing rules. Repetitive or low-value procedures should be simplified or removed, while necessary accountability requirements should be retained. Digital technologies may also be used to integrate information systems and reduce repetitive reporting, provided that digitalization does not create additional procedural burdens.

Finally, managers should recognize that strengthening assessment alone may be insufficient when employees operate under high levels of red tape. Reform efforts should simultaneously improve performance management and reduce unnecessary administrative burdens. This coordinated approach can provide village-assigned officials with clearer institutional guidance while preserving the time, autonomy, and psychological resources needed to serve rural communities effectively.

## 8 Limitations and future research

Despite the theoretical and practical implications of the findings, the current study is not free of limitations which should be addressed in subsequent researches. First, because we obtained the data from a sample of public servants in China, the findings might not be generalizable to public servants in other countries. Further investigation is needed to enhance the generalizability of the findings to other countries and industry. Second, the present study used a cross-sectional design; thus, it may preclude coming to any causal conclusions. In future research, more rigorous research designs, such as experimental or longitudinal design, will be needed to facilitate the causality of the relationships among variables. A suggestion for future research direction is to look for other variables that may serve as moderators in the relationship between SAM and PSM. For example, personality traits (e.g., emotional intelligence, proactive personality and core self-evaluations, etc.) and contextual factors (e.g., leader-member exchange, perceived organizational support, etc.) are likely to be good candidates for moderators. In addition, although CMB was not found to be a major problem to the current study, multiple sources for the data would strengthen research validity. For instance, public servants’ work performance could be evaluated by their supervisors or organization’s work performance data archives(Min et al., 2015).

## 9 Conclusion

Based on a sample of 354 Chinese village-assigned officials, this study proposes and tests a moderated mediation model in which the indirect effect of SAM on work performance through PSM is moderated by red tape. The results reveal that PSM mediates the relationship between SAM and work performance. The results further demonstrates that the indirect effect is at its highest when red tape is low. This means that the positive indirect effect of SAM is not equal for all public servants, rather depends upon the level of public servants’ red tape. The main message from these findings is that public organizations should endeavor to diminish red tape public servants feel and experience in the workplace. We hope that the present study will add value to the current literature regarding the research on PSM and work performance.

## Data Availability

The raw data supporting the conclusions of this article will be made available by the authors, without undue reservation.

## References

[ref1] Bandura A. (1986). Social Foundations of Thought and Action: A Social Cognitive Theory. Englewood Cliffs: Prentice-Hall.

[ref2] Bao Y. J. Li C. P. (2016). Measuring public service motivation: theoretical structure and scale revision. Hum. Resour. Dev. China 7, 83–91. doi: 10.16471/j.cnki.11-2822/c.2016.07.010

[ref3] Blom R. Borst R. T. Voorn B. (2021). Pathology or inconvenience? A meta-analysis of the impact of red tape on people and organizations. Rev. Public Pers. Adm. 41, 623–650. doi: 10.1177/0734371X20924117

[ref4] Bozeman B. Feeney M. K. (2014). Rules and Red Tape: A Prism for Public Administration Theory and Research. New York: Routledge.

[ref5] Bright L. (2007). Does person-organization fit mediate the relationship between public service motivation and the job performance of public employees? Rev. Public Pers. Adm. 27, 361–379. doi: 10.1177/0734371X07307149

[ref6] Carver C. S. Scheier M. F. (1982). Control theory: a useful conceptual framework for personality-social, clinical, and health psychology. Psychol. Bull. 92, 111–135. doi: 10.1037/0033-2909.92.1.111, 7134324

[ref7] Cheung G. W. Lau R. S. (2017). Accuracy of parameter estimates and confidence intervals in moderated mediation models: a comparison of regression and latent moderated structural equations. Organ. Res. Methods 20, 746–769. doi: 10.1177/1094428115595869

[ref8] Chung K. H. Rhee I. Liu C. (2024). A systematic review of experimental research on public service motivation. Public Perform. Manag. Rev. 47, 627–653. doi: 10.1080/15309576.2023.2269386

[ref9] DeNisi A. S. Pritchard R. D. (2006). Performance appraisal, performance management, and improving individual performance: a motivational framework. Manag. Organ. Rev. 2, 253–277. doi: 10.1111/j.1740-8784.2006.00042.x

[ref10] Farh J. L. Cannella A. A. Lee C. (2006). Approaches to scale development in Chinese management research. Manag. Organ. Rev. 2, 301–318. doi: 10.1111/j.1740-8784.2006.00055.x

[ref11] Fornell C. Larcker D. F. (1981). Evaluating structural equation models with unobservable variables and measurement error. J. Mark. Res. 18, 39–50. doi: 10.2307/3151312

[ref12] Fuenzalida J. Gutiérrez L. L. Fernández-Vergara A. González P. A. (2026). Red tape and burnout risks in the public service: evidence from a survey experiment of school principals. Rev. Public Pers. Adm. 46, 53–84. doi: 10.1177/0734371X241261083

[ref13] George B. Pandey S. K. Steijn B. Decramer A. Audenaert M. (2021). Red tape, organizational performance, and employee outcomes: meta-analysis, meta-regression, and research agenda. Public Adm. Rev. 81, 638–651. doi: 10.1111/puar.13327

[ref14] Gjersing L. Caplehorn J. R. M. Clausen T. (2010). Cross-cultural adaptation of research instruments: language, setting, time and statistical considerations. BMC Med. Res. Methodol. 10:13. doi: 10.1186/1471-2288-10-13, 20144247 PMC2831007

[ref15] Guo Q. Q. Guo C. F. (2019). Antecedent variables, dimensional testing, and measurement of public service motivation among civil servants in western China: evidence from a survey of MPA students at six universities in western China. Leadersh. Sci. Forum 11, 32–52. doi: 10.19299/j.cnki.42-1837/c.2019.11.003

[ref16] Hair J. F. Black W. C. Babin B. J. Anderson R. E. (2010). Multivariate Data Analysis. 7th Edn Upper Saddle River: Pearson Prentice Hall.

[ref17] Han Q. Y. (2021). The Driving Effect of Public Service Motivation on Job Performance Among Civil Servants in a Collectivist Cultural Context (master’s thesis). Tianjin: Nankai University.

[ref18] Han Y. Liao J. Q. Long L. R. (2007). Model of development and empirical study on employee job performance construct. J. Manag. Sci. China 10, 62–77. doi: 10.3321/j.issn:1007-9807.2007.05.007

[ref19] Hayes A. F. (2015). An index and test of linear moderated mediation. Multivar. Behav. Res. 50, 1–22. doi: 10.1080/00273171.2014.962683, 26609740

[ref20] Hayes A. F. (2022). Introduction to Mediation, Moderation, and Conditional Process Analysis. 3rd Edn New York, NY: Guilford Press.

[ref21] Hobfoll S. E. (1989). Conservation of resources: a new attempt at conceptualizing stress. Am. Psychol. 44, 513–524. doi: 10.1037/0003-066X.44.3.513, 2648906

[ref22] Hobfoll S. E. Halbesleben J. Neveu J. P. Westman M. (2018). Conservation of resources in the organizational context: the reality of resources and their consequences. Annu. Rev. Organ. Psychol. Organ. Behav. 5, 103–128. doi: 10.1146/annurev-orgpsych-032117-104640

[ref23] Homans G. C. (1958). Social behavior as exchange. Am. J. Sociol. 63, 597–606. doi: 10.1086/222355

[ref24] Huang Q. Zhang K. Bodla A. A. Wang Y. (2022). The influence of perceived red tape on public employees’ procrastination: the conservation of resource theory perspective. Int. J. Environ. Res. Public Health 19:4368. doi: 10.3390/ijerph19074368, 35410047 PMC8999032

[ref25] Jia X. D. (2010). A Grounded Theory Study of Public Service Outsourcing by Urban Grassroots Governments in China (Dissertation). Lanzhou: Lanzhou University.

[ref26] Jung C. S. (2014). Why are goals important in the public sector? Exploring the benefits of goal clarity for reducing turnover intention. J. Public Adm. Res. Theory 24, 209–234. doi: 10.1093/jopart/mus058

[ref27] Kim S. (2004). Individual-level factors and organizational performance in government organizations. J. Public Adm. Res. Theory 15, 245–261. doi: 10.1093/jopart/mui013

[ref28] Klein A. Moosbrugger H. (2000). Maximum likelihood estimation of latent interaction effects with the LMS method. Psychometrika 65, 457–474. doi: 10.1007/BF02296338

[ref29] Kline R. B. (2016). Principles and Practice of Structural Equation Modeling. 4th Edn New York: Guilford Press.

[ref30] Li S. S. (2023). A Study on the Optimization of Performance Evaluation for Digital Urban Management in Quanzhou (Master’s thesis). Quanzhou: Huaqiao University.

[ref31] Li F. Wang P. Q. (2016). Structure and antecedents of public service motivation among grassroots civil servants. J. Central China Normal Univ. Humanit. Soc. Sci. 55, 29–38.

[ref32] Liao X. M. (2021). Work Stress and Job Performance Among Grassroots Discipline Inspection and Supervision Cadres in Ya’an City (Master’s thesis). Chengdu: Sichuan University.

[ref33] Locke E. A. Latham G. P. (1990). A Theory of Goal Setting & Task Performance. Englewood Cliffs: Prentice Hall.

[ref34] Mao H. P. (2018). Research on the Management of Village-Stationed Cadres in the Context of Poverty Alleviation: A Case Study of S City (Master’s thesis). Xi’an: Northwest University.

[ref35] Min H. Kim H. J. Lee S.-B. (2015). Extending the challenge-hindrance stressor framework: the role of psychological capital. Int. J. Hosp. Manag. 50, 105–114. doi: 10.1016/j.ijhm.2015.07.006

[ref36] Moynihan D. P. Pandey S. K. (2007). The role of organizations in fostering public service motivation. Public Adm. Rev. 67, 40–53. doi: 10.1111/j.1540-6210.2006.00695.x

[ref37] Pandey S. K. (2021). The psychological process view of bureaucratic red tape. In: Research Handbook on HRM in the Public Sector, eds. Knies E. Steijn B.. Edward Elgar. 260–275. doi: 10.4337/9781789906622.00028

[ref001] Pandey S. K. Scott P. G. (2002). Red tape: a review and assessment of concepts and measures. J. Public Adm. Res. Theory 12, 553–580. doi: 10.1093/oxfordjournals.jpart.a003547

[ref38] Perry J. L. (1996). Measuring public service motivation: an assessment of construct reliability and validity. J. Public Adm. Res. Theory 6, 5–22. doi: 10.1093/oxfordjournals.jpart.a024303

[ref39] Perry J. L. Hondeghem A. Wise L. R. (2010). Revisiting the motivational bases of public service: twenty years of research and an agenda for the future. Public Adm. Rev. 70, 681–690. doi: 10.1111/j.1540-6210.2010.02196.x

[ref40] Perry J. L. Wise L. R. (1990). The motivational bases of public service. Public Adm. Rev. 50, 367–373. doi: 10.2307/976618

[ref41] Podsakoff P. M. MacKenzie S. B. Lee J.-Y. Podsakoff N. P. (2003). Common method biases in behavioral research: a critical review of the literature and recommended remedies. J. Appl. Psychol. 88, 879–903. doi: 10.1037/0021-9010.88.5.879, 14516251

[ref42] Qiang Q. Wang K. Lai J. (2025). Does the perception of red tape affect the emotional labor of frontline retail staff in China? A post-COVID-19 era. PLoS One 20:e0327359. doi: 10.1371/journal.pone.0327359, 40674307 PMC12270127

[ref43] Ritz A. Brewer G. A. Neumann O. (2016). Public service motivation: a systematic literature review and outlook. Public Adm. Rev. 76, 414–426. doi: 10.1111/puar.12505

[ref44] Ritz A. Vandenabeele W. Vogel D. (2021). “Public service motivation and individual job performance,” in Managing for Public Service Performance: How People and Values Make a Difference, eds. Leisink P. Andersen L. B. Brewer G. A. Jacobsen C. B. Knies E. Vandenabeele W. (Oxford: Oxford University Press), 254–277.

[ref45] Sardeshmukh S. R. Vandenberg R. J. (2017). Integrating moderation and mediation: a structural equation modeling approach. Organ. Res. Methods 20, 721–745. doi: 10.1177/1094428115621609

[ref46] Su W. W. (2023). A Performance Appraisal System for W Bank Based on the Balanced Scorecard (Master’s thesis). Hangzhou: Zhejiang Sci-Tech University.

[ref47] Vandenabeele W. (2007). Toward a public administration theory of public service motivation: an institutional approach. Public Manag. Rev. 9, 545–556. doi: 10.1080/14719030701726697

[ref48] Wang P. P. (2023). Professional Identity and Job Performance Among Public Hospital Physicians: The Mediating Role of Public Service Motivation (Master’s thesis). Jinan: Shandong University.

[ref49] Wen B. Tao L. (2022). Public service motivation in the Chinese context: theory construction and workplace consequences. Adv. Psychol. Sci. 30, 239–254. doi: 10.3724/SP.J.1042.2022.00239

[ref50] Wright B. E. Grant A. M. (2010). Unanswered questions about public service motivation: designing research to address key issues of emergence and effects. Public Adm. Rev. 70, 691–700. doi: 10.1111/j.1540-6210.2010.02197.x

[ref51] Yu W. D. (2023). Effects of Public Service Motivation on Job Performance Among Township Civil Servants (Master’s thesis). Nanning: Guangxi University.

[ref52] Zhang Q. (2022). Public Service Motivation and Job Performance Among Civil Servants (Master’s thesis). Yan’an: Yan’an University.

[ref53] Zhao S. Y. (2022). Red Tape and Organizational Citizenship Behavior Among Civil Servants (Master’s thesis). Chengdu: Southwestern University of Finance and Economics.

[ref54] Zhou C. (2018). Research on Factors Influencing Executive Capability in Large Construction Enterprises (Master’s thesis). Xi’an: Xi’an University of Science and Technology.

